# Dexamethasone and Monophosphoryl Lipid A-Modulated Dendritic Cells Promote Antigen-Specific Tolerogenic Properties on Naive and Memory CD4^+^ T Cells

**DOI:** 10.3389/fimmu.2016.00359

**Published:** 2016-09-19

**Authors:** Jaxaira Maggi, Katina Schinnerling, Bárbara Pesce, Catharien M. Hilkens, Diego Catalán, Juan C. Aguillón

**Affiliations:** ^1^Programa Disciplinario de Inmunología, Immune Regulation and Tolerance Research Group, Facultad de Medicina, Instituto de Ciencias Biomédicas (ICBM), Universidad de Chile, Santiago, Chile; ^2^Millennium Institute on Immunology and Immunotherapy (MIII), Santiago, Chile; ^3^Musculoskeletal Research Group, Faculty of Medical Sciences, Institute of Cellular Medicine, Newcastle University, Newcastle upon Tyne, UK

**Keywords:** tolerance, monocyte-derived dendritic cells, hyporesponsiveness, naive CD4^+^ T cells, memory CD4^+^ T cells, immunotherapy

## Abstract

Tolerogenic dendritic cells (DCs) are a promising tool to control T cell-mediated autoimmunity. Here, we evaluate the ability of dexamethasone-modulated and monophosphoryl lipid A (MPLA)-activated DCs [MPLA-tolerogenic DCs (tDCs)] to exert immunomodulatory effects on naive and memory CD4^+^ T cells in an antigen-specific manner. For this purpose, MPLA-tDCs were loaded with purified protein derivative (PPD) as antigen and co-cultured with autologous naive or memory CD4^+^ T cells. Lymphocytes were re-challenged with autologous PPD-pulsed mature DCs (mDCs), evaluating proliferation and cytokine production by flow cytometry. On primed-naive CD4^+^ T cells, the expression of regulatory T cell markers was evaluated and their suppressive ability was assessed in autologous co-cultures with CD4^+^ effector T cells and PPD-pulsed mDCs. We detected that memory CD4^+^ T cells primed by MPLA-tDCs presented reduced proliferation and proinflammatory cytokine expression in response to PPD and were refractory to subsequent stimulation. Naive CD4^+^ T cells were instructed by MPLA-tDCs to be hyporesponsive to antigen-specific restimulation and to suppress the induction of T helper cell type 1 and 17 responses. In conclusion, MPLA-tDCs are able to modulate antigen-specific responses of both naive and memory CD4^+^ T cells and might be a promising strategy to “turn off” self-reactive CD4^+^ effector T cells in autoimmunity.

## Introduction

One of the main challenges in the field of autoimmunity is the attenuation of aberrant T cell immune responses to “self.” Current treatments, such as immunosuppressive drugs, evoke undesired side effects and an increased probability of acquiring infectious diseases and cancer (1, 2). In this context, a novel and promising immunotherapeutic strategy to specifically control T cell-mediated pathologies is the use of *ex vivo*-modulated dendritic cells (DCs) (3).

Dendritic cells have been described as the most potent antigen-presenting cells and are fundamental for the initiation of an effective immune response (4). Depending on their maturation stage, DCs are able to induce or inhibit T cell responses (5): while immature and semi-mature DCs are considered tolerogenic (tDCs), mature DCs (mDCs) are immunogenic. tDCs exhibit a reduced costimulatory capacity and an anti-inflammatory cytokine secretion profile and are able to modulate CD4^+^ T cell responses (6, 7). The mechanisms by which tDCs exert their modulatory activity on autoreactive T cells include clonal deletion, anergy induction, or cell reprograming. Additionally, tDCs are able to promote the differentiation and proliferation of T cells with regulatory functions (Treg) (8).

It has been reported that tDCs have great therapeutic potential. Injection of *ex vivo* modified tDCs has provided improvement in murine models of autoimmune diseases, including arthritis (9–12), diabetes (13, 14), and multiple sclerosis (15). In humans, phase I clinical trials using tDCs have been carried out in patients with type 1 diabetes (16) and rheumatoid arthritis (17, 18). In all cases, treatment was well tolerated by patients without side effects, justifying further studies to evaluate their clinical efficacy and antigen-specific impact.

There are different methods for *in vitro* generation of tDCs from peripheral blood monocytes (19), such as genetic modification (20–22), pharmacological modulation (e.g., with vitamin D3, dexamethasone, or rapamycin) (6, 23, 24), or treatment with anti-inflammatory cytokines, IL-10 or TGF-β (25). It has been described that alternative activation of tDCs, induced by proinflammatory mediators, such as TNF-α, IL-1, and IL-6, or toll-like receptor ligands, such as LPS, improves their antigen-presenting capacity and endows them with the ability to migrate to secondary lymphoid organs (26–28).

Recently, we described a 5-day protocol for the generation of stable semi-mature monocyte-derived tDCs using dexamethasone (Dex), as immunomodulatory agent, and monophosphoryl lipid A (MPLA), a non-toxic (GMP-compatible) LPS analog, as activating stimulus (MPLA-tDCs). Similar to Dex-modulated tDCs, which have been well described as tolerogenic, these MPLA-tDCs are characterized by a reduced expression of costimulatory molecules (CD80, CD86, and CD40), an IL-10^high^/IL-12^low^ cytokine secretion profile, and a reduced ability to stimulate proliferation and proinflammatory cytokine secretion of allogeneic and antigen-specific CD4^+^ T cells. Importantly, the activation of MPLA-tDCs using MPLA upregulates expression of CCR7 and CXCR4 chemokine receptors in comparison to tDCs, conferring to MPLA-tDCs the lymph node homing-capacity, which together with their potential to induce high levels of IL-10 secretion in co-cultures with CD4^+^ T cells suggests that MPLA-tDCs might be superior to Dex-modulated tDCs regarding location for interacting with autoreactive effector CD4^+^ T cells and subsequent tolerance recovery (26).

To validate the suitability of MPLA-tDCs for autologous immunotherapy of autoimmune disorders, it is crucial to confirm their ability to act at different levels of an immune response, either by directing differentiation of naive CD4^+^ T cells with certain antigen-specificity toward a regulatory profile or by reprograming autoreactive memory CD4^+^ T cells. Different studies reported the effects of Dex-modulated tDCs on CD4^+^ T cell subsets in allogeneic models, with controversial conclusions. It has been described that both naive and memory CD4^+^ T cells primed by Dex-modulated tDCs become hyporesponsive upon restimulation with mDCs *via* the induction of anergy (29). Other studies showed that tDCs generated with Dex alone, or in combination with vitamin D3 and LPS, polarize naive CD4^+^ T cells toward Treg cells with an IFNγ^low^/IL-10^high^ cytokine profile, while rendering memory CD4^+^ T cells anergic (27).

In this work, we investigated the modulation of antigen-specific naive and memory CD4^+^ T cell responses by MPLA-tDCs to get further insight into their immunomodulatory mechanisms. We demonstrate that MPLA-tDCs display a reduced ability to induce proliferation and proinflammatory cytokine production of CD4^+^ memory T cells and promote hyporesponsiveness to restimulation. Furthermore, we show that MPLA-tDCs are capable of instructing naive CD4^+^ T cells at the priming, reducing proliferation and secretion of proinflammatory cytokines in response to restimulation and conferring them the ability to suppress T helper type 1 (Th1) and Th17 responses. This confirms that MPLA-tDCs are able to reprogram antigen-specific naive and memory CD4^+^ T cell responses.

## Materials and Methods

### Samples and Isolation of Cell Populations

Buffy coats from healthy donors were obtained from the Blood Bank of Clinical Hospital from Universidad de Chile. All donors had been vaccinated with Bacillus Calmette–Guérin (BCG), resulting in T cell reactivity against purified protein derivative (PPD) antigen. CD14^+^ monocytes were obtained by negative selection using RosetteSep^®^ Human Monocyte Enrichment Cocktail (Stem Cell Technologies, Vancouver, BC, Canada), followed by density gradient centrifugation with Lymphoprep (Axis-Shield Diagnostics, Dundee, UK). CD45RA^+^/RO^−^ naive and CD45RA^–^/RO^+^ memory CD4^+^ T cells were purified from PBMC by negative magnetic selection using EasySep™ Human Naive or Memory CD4^+^ T Cell Enrichment Kits (StemCell Technologies), respectively.

Subjects signed an informed written consent according to the Declaration of Helsinki, and all procedures were approved by the Ethics Committees of the Faculty of Medicine and the Clinical Hospital of University of Chile.

### Generation of Dendritic Cells

Dendritic cells were generated from monocytes and cultured at 2–3 × 10^6^ cells/ml in AIM-V serum-free medium (GIBCO BLR, Invitrogen, Grand Island, USA), in the presence of rhIL-4 and rhGM-CSF (500 U/ml each; eBioscience, San Diego, CA, USA) for 5 days at 37°C and 5% CO_2_ (26). Activated tDCs (MPLA-tDCs) were generated by addition of 1 μM Dex (Sigma-Aldrich, St. Louis, MO, USA) at day 3 and 1 μg/ml MPLA (Avanti Polar Lipids Inc., Alabaster, AL, USA) 24 h later. Three controls were used: untreated immature cells (iDCs), tolerogenic Dex-treated DCs (tDCs), and mature MPLA-treated DCs (mDCs). On day 5, cells were harvested and phenotypic and functional analyses were performed.

### Flow Cytometry Analysis

The following antibodies were used for flow cytometry analysis: CD11c APC, CD80 FITC, CD83 FITC, CD86 PE, HLA-DR FITC, CD1a FITC, CD14 FITC, CD4PE, CD4 PE-Cy7, CD45RA PE, CD45RO FITC, CD25 PE-Cy7, CTLA-4 APC, CD39 PerCP-eFluor710, FOXP3 Alexa Fluor 700, IL-10 PE, IFN-γ PE-Cy7, and IL-17 APC (all eBioscience). For surface staining, cells were incubated in PBS 10% FBS containing the respective antibodies for 30 min at 4°C, washed, and stored in IC fixation buffer until analysis (eBioscience). Intracellular FoxP3 was detected using FoxP3-staining kit (eBioscience) according to the manufacturer’s instructions. For intracellular cytokines detection, cells were treated with 50 ng/ml PMA, 1 μg/ml ionomycin, and 1 μl/ml brefeldin A for 5 h. After harvesting, cells were surface stained for CD4. Intracellular cytokine staining was performed in permeabilization buffer (eBioscience) before cells were washed and resuspended in FACS buffer for flow cytometry analysis. Cell viability was assessed by 7-AAD and annexin-V PE staining (eBioscience). Data were collected on FACSCalibur and FACSAria cytometers (Beckton Dickinson, San Diego, CA, USA) and analyzed with Weasel v3.0.2 software.

### Antigen Uptake Assay

Dendritic cells were incubated with 1 mg/ml of dextran (DX)-FITC (40 kDa; Molecular Probes, Leiden, Netherlands) for 1, 2, and 4 h at 37 or 4°C (negative control). Uptake was stopped with cold PBS, and samples were analyzed by flow cytometry. Endocytosis capacity is represented by the difference between FITC mean fluorescence intensity (MFI) of cells incubated at 37°C and cells incubated at 4°C.

### Antigen Presentation Assay

Different subtypes of DCs were generated from HLA DRB1*0101 donors, as described above. On day 4, DCs were loaded with human type II collagen peptide hCII^259–273^ or an irrelevant chicken peptide, 6 h before addition of MPLA. Unloaded DCs were used as control. On day 5, DCs were fixed with paraformaldehyde and co-cultured with the transgenic murine HLA-DR1-restricted T cell hybridoma HCII-9.1, specific for the non-glycosylated version of the immunodominant T cell epitope of hCII (hCII^259–273^) (30). Co-culture was performed in RPMI medium supplemented with 10% FBS at 1:10 ratio (DCs/HCII-9.1) for 24 h. Supernatants were recovered and IL-2 secretion by the T cell hybridoma was determined based on the proliferative response of the IL-2-dependent cytotoxic T cell line-2 (CTLL-2). CTLL-2 cells were labeled with 5 μM carboxyfluorescein succinimidyl ester (CFSE; Sigma-Aldrich), stimulated with co-culture supernatants for 24 h, and their proliferation was assessed through dilution of CFSE fluorescence by flow cytometry.

### Antigen-Specific T Cell Stimulation and Restimulation Assays

On day 4 of DC generation, 4 h before stimulation with MPLA, DCs were loaded with 1 μg/ml PPD (Staten Serum Institute, Copenhagen, Denmark), or left unloaded (controls for unspecific stimulation), and incubated for further 24 h. DCs were harvested, washed, and co-cultured with CFSE-labeled (primary T cell stimulation assay) or unlabeled (restimulation assay) autologous naive or memory CD4^+^ T cells at a ratio of 1:10 (DC/naive T cells) and 1:5 (DC/memory T cells) in RPMI medium supplemented with 10% FBS, for 6 days. CD4^+^ T cells alone or stimulated with plate-bound anti-human CD3 antibody (OKT3; 0.65 μg/well; eBioscience) were used as negative and positive controls for T cell activation, respectively.

For restimulation assays, naive or memory CD4^+^ T cells were recovered from initial co-cultures, extensively washed, labeled with CFSE, and restimulated with PPD-loaded mDCs from the same donor, at a DC/T cell ratio of 1:5. Supernatants were collected after 5 days of restimulation and assayed for IL-10 by ELISA. T cell proliferation, intracellular cytokine expression, and regulatory phenotype markers were assessed by flow cytometry.

### T Cell Suppression Assay

Naive CD4^+^ T cells primed with different PPD-loaded DC subtypes as described above were recovered, washed, and co-cultured with autologous CFSE-labeled responder CD4^+^ T cells and PPD-loaded mDCs at a primed-naive T cells/responder CD4^+^ T cells/mDCs ratio of 1:1:1. After 5 days, proliferation and intracellular cytokine expression of responder CD4^+^ T cells were determined by flow cytometry.

### Statistical Analyses

GraphPad Prism version 5.0 (GraphPad Software, San Diego, CA, USA) was used for statistical analyses and graph preparation. Data distribution was analyzed by Kolmogorov–Smirnov test. According to the results of the normality test, Friedman test (non-parametric data) or one-way ANOVA (normally distributed data) for repeated measures, followed by Dunns or Tukey post-test, respectively, were used for statistical comparisons. For grouped analyses, two-way ANOVA and Bonferroni post-tests were applied to compare replicate means by row. *P* values ≤0.05 were considered significant.

## Results

### MPLA-tDCs Are Immature Dendritic Cells That Present Antigen Efficiently

**Figure 1:**
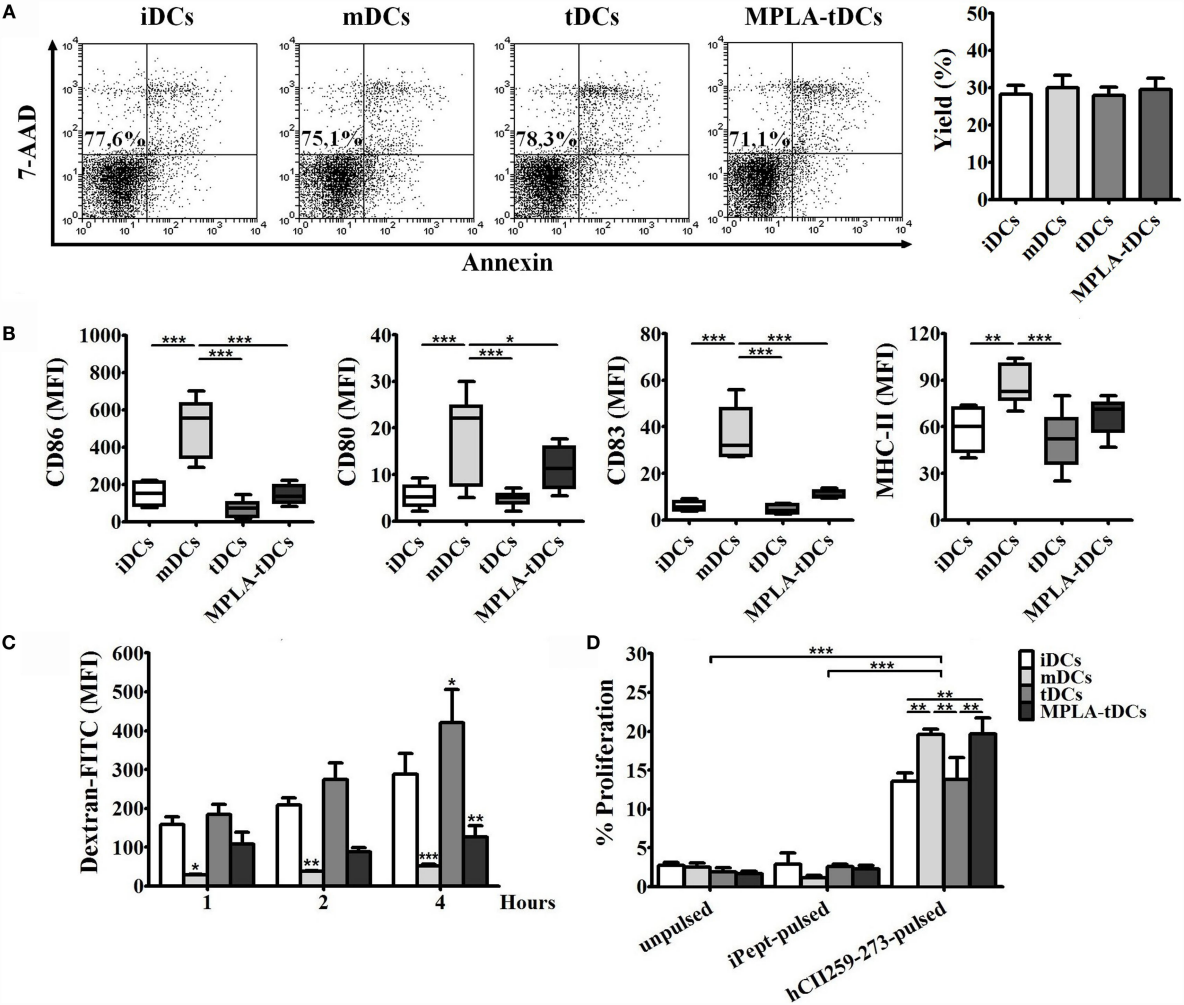
**MPLA-tDCs are characterized by low expression of maturation markers, diminished endocytosis ability, and the capability to present antigen efficiently**. For the induction of a tolerogenic state, DCs were conditioned with dexamethasone (Dex) and were additionally activated with MPLA (MPLA-tDCs). Untreated DCs (iDCs), Dex-modulated DCs (tDCs), and MPLA-matured DCs (mDCs) were used as controls. **(A)** Representative dot plots of cell viability measured after 5 days of culture and expressed as the percentage of annexin-V and 7-AAD-negative cells are shown, and DC yield is expressed as a percentage of DCs obtained on day 5 related to the initial number of monocytes cultured per condition (mean ± SEM) (*n* = 10). **(B)** Surface expression levels of CD86, CD80, CD83, and HLA-DR were assessed by flow cytometry. Graphic analyses of MFI measurements are expressed as box-and-whiskers plot from minimum to maximum values (*n* = 6). Statistical differences were calculated using one-way ANOVA for repeated measures followed by Tukey post-test (*P < 0.05; **P < 0.01; ***P < 0.001). **(C)** Endocytosis capacity was assessed by incubating DCs with dextran-FITC during 1, 2, and 4 h. Results are expressed as the difference between MFI of cells incubated at 37 and 4°C with the antigen and are represented as the mean ± SEM of four independent experiments. Significant differences are expressed in relation to iDCs condition. **(D)** For evaluating antigen-presentation ability, DCs generated from HLA-DRB1*0101 donors were pulsed with the human type II collagen peptide (hCII259–273) and co-cultured with the HLA-DR1-restricted T cell hybridoma (hCII-9.1). Unpulsed DCs and DCs loaded with an irrelevant chicken peptide (iPept) were used as controls. Graphs show the proliferative responses of CTLL-2 cells incubated with supernatants obtained from those co-cultures and assessed by flow cytometry. Data are represented as the mean ± SEM of three independent experiments. For **(C,D)**, statistical differences were calculated using two-way ANOVA followed by Bonferroni post-test (*P < 0.05; **P < 0.01; ***P < 0.001).

MPLA-tDCs, generated using a combination of Dex and MPLA, were phenotypically and functionally characterized by flow cytometry in comparison to untreated iDCs, MPLA-matured mDCs, and Dex-modulated tDCs. All DC subtypes comprised >95% CD11c^+^ cells with a CD1a^high^ and CD14^low^ phenotype, and DC viability, assessed by 7-AAD and annexin-V staining, was not affected by treatment with Dex or MPLA (Figure 1A). Phenotypic analysis revealed that MPLA-tDCs exhibited low expression levels of the costimulatory molecules CD86 and CD80 and the maturation marker CD83, similar to iDCs and tDCs, but significantly reduced in comparison to mDCs. Concerning the human leukocyte antigen HLA-DR, MPLA-tDCs did not present significant differences in expression levels as compared to mDCs (Figure 1B).

To determine the capacity of MPLA-tDCs to pick up antigen, endocytosis of FITC-conjugated dextran particles was determined by flow cytometry and compared between different DC preparations. While iDCs and tDCs displayed a potent endocytic ability that increased with time, MPLA treatment (mDCs and MPLA-tDCs) markedly reduced uptake of DX-FITC by DCs, especially after prolonged incubation (Figure 1C). Based on these results, to favor endocytosis in subsequent assays, DCs were pulsed with the antigen for 4 h before being activated with MPLA.

To evaluate if MPLA-tDCs are capable of presenting antigen peptides to T cells, DC preparations, generated from HLA DRB1*0101 donors and pulsed with the collagen peptide hCII_259–273_, or an irrelevant chicken-derived peptide, were co-cultured with the HLA-DR-restricted T cell hybridoma HCII-9.1. Recognition of the presented hCII_259–273_ peptide, but not for the irrelevant peptide, by hybridoma cells, induced IL-2 secretion into culture supernatants, which in turn stimulated the proliferation of the IL-2-dependent cell line CTLL-2 (Figure 1D). Significant differences were observed between percentages of CTLL-2 proliferation induced by IL-2 coming from co-cultures performed with mDCs and MPLA-tDCs compared to the other conditions (Figure 1D). Proliferative responses of CTLL-2 revealed that activation with MPLA increase the ability of tDCs to present the hCII_259–273_ peptide efficiently.

### MPLA-tDCs Modulate Responses of Antigen-Specific CD4^+^ Memory T Cells

**Figure 2:**
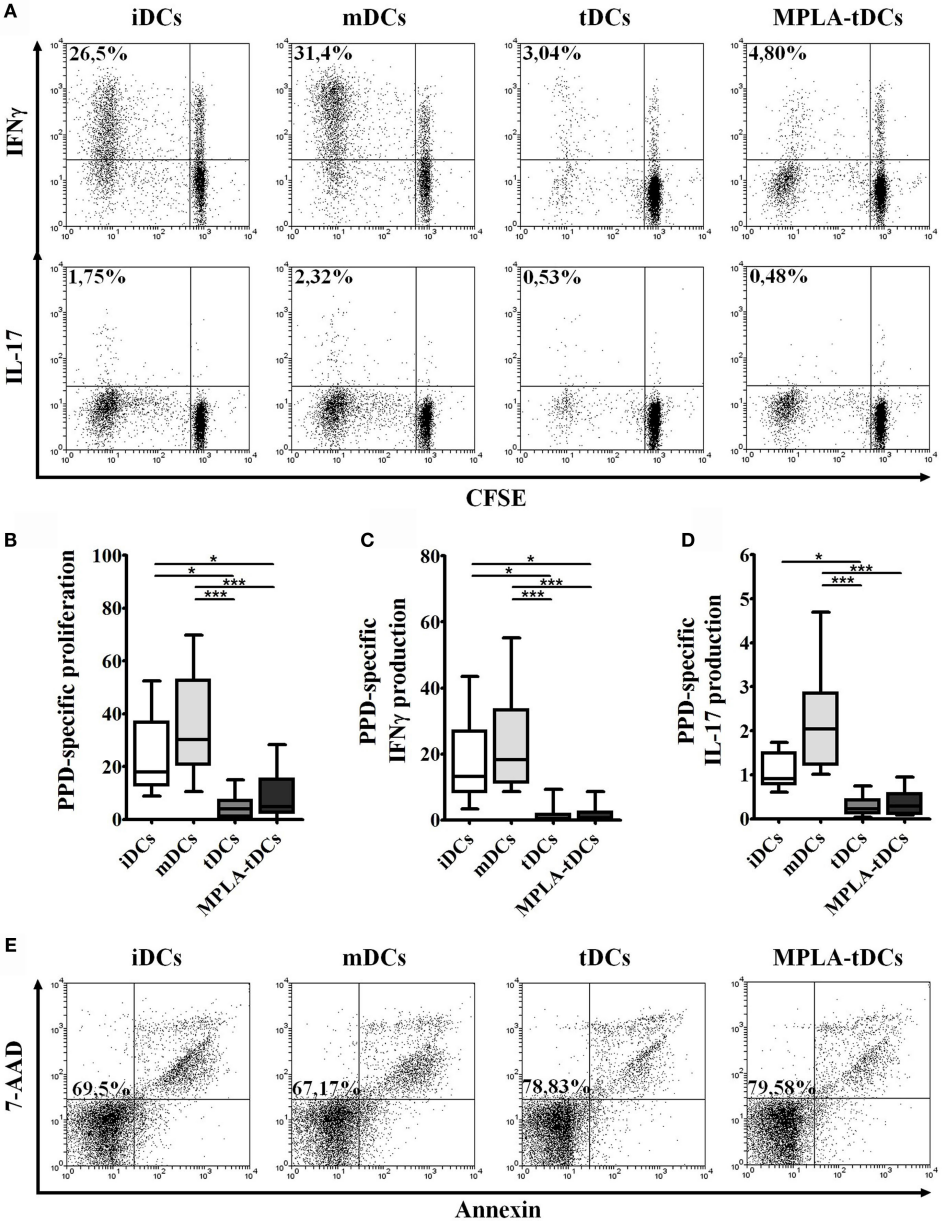
**MPLA-tDCs modulate memory CD4^+^ T cells responses in an antigen-specific way**. For the assessment of their T cell-stimulatory capacity, PPD-pulsed DCs were co-cultured with autologous CFSE-labeled memory CD4^+^ T cells. Un-loaded DCs were used as control. Data were normalized by subtracting values of unpulsed conditions to antigen-specific conditions. **(A)** Representative dot plots of IFN-γ (top) and IL-17 (bottom) expression from 10 independent experiments are shown. **(B)** Proliferation of CD4^+^ T cells was determined by CFSE dilution through flow cytometry, and graphic representation of normalized data of the percentage of CFSE^low^ in CD4^+^ T cells is shown. **(C)** IFN-γ and **(D)** IL-17 expression was detected intracellularly and analyzed by flow cytometry. Graphic representation of normalized data of the percentage of IFN-γ and IL-17 producing proliferating CD4^+^ T cells (CFSE^low^) is shown. In **(B–D)**, statistical differences were calculated using Friedman test followed by Duns post-test (*P < 0.05; ***P < 0.001). **(E)** Representative dot plots of cell viability measured after 6 days of co-culture and expressed as the percentage of annexin-V and 7-AAD-negative cells are shown.

To determine the capacity of MPLA-tDCs to stimulate memory T cell responses, DC preparations, either loaded with PPD or unloaded, were co-cultured with autologous memory CD4^+^ T cells, and proliferation and cytokine production were assessed. A reduced proliferative response and proinflammatory cytokines production were evidenced when memory CD4^+^ T cells were co-cultured with different unpulsed DC preparations in comparison to pulsed conditions (data not shown). PPD-pulsed mDCs were potent inducers of memory CD4^+^ T cell proliferation. In contrast, the immunostimulatory capacity of tDCs and MPLA-tDCs was even lower than that of iDCs (Figure 2B). To further characterize the type of antigen-specific response induced by each DC subset, intracellular expression of IFN-γ and IL-17 by memory CD4^+^ T cells was assessed (Figure 2A). Both PPD-pulsed iDCs and mDCs induced IFN-γ expression in a significantly higher percentage of proliferating memory CD4^+^ T cells than PPD-pulsed tDCs or MPLA-tDCs (Figure 2C). IL-17-producing proliferating memory CD4^+^ T cells increased significantly in response to stimulation with PPD-pulsed mDCs in comparison to PPD-pulsed tDCs or MPLA-tDCs (Figure 2D). Antigen-pulsed tDCs and MPLA-tDCs were even poorer inducers of proinflammatory cytokine responses in memory CD4^+^ T cells than iDCs (Figures 2B,C). A combined staining of annexin-V and 7-AAD evidenced that the treatment applied to DCs did not affect the viability of memory CD4^+^ T cells in the co-cultures (Figure 2E).

### Naive CD4^+^ T Cells Primed by MPLA-tDCs Do Not Exhibit a Regulatory Phenotype

In order to examine the ability of MPLA-tDCs to modulate naive CD4^+^ T cell responses, PPD-pulsed or unpulsed DC preparations were co-cultured with autologous naive CD4^+^ T cells. After 6 days of co-culture, no proliferative responses of naive CD4^+^ T cells were observed, irrespective of whether they were primed by PPD-loaded iDCs, mDCs, tDCs, or MPLA-tDCs (data not shown). The lack of proliferation was not due to apoptosis as confirmed by annexin-V and 7-AAD staining (Figure 4B).

To investigate if MPLA-tDCs are able to induce phenotypic alterations in naive CD4^+^ T cells or imprint regulatory features, the expression of IL-10 and regulatory T cell markers was evaluated. Surprisingly, the percentage of Foxp3^+^ CD25^hi^ cells was significantly lower when naive CD4^+^ T cells were primed by MPLA-tDCs or tDCs, compared to when iDCs or mDCs were used as stimulators (Figure 4A). Likewise, the expression of the co-inhibitory receptor CTLA-4 and the ectonucleotidase CD39, both involved in regulatory functions, was also reduced in CD4^+^ T cells primed by MPLA-tDCs or tDCs as compared to iDCs- or mDCs-primed CD4^+^ T cells (Figure 4A). There were also no significant differences regarding the expression of IL-10 between CD4^+^ T cells primed by MPLA-tDCs, tDCs, iDCs, or mDCs (Figure 4A).

### MPLA-tDCs Render Naive and Memory CD4^+^ T Cells Hyporesponsive to Restimulation

**Figure 3:**
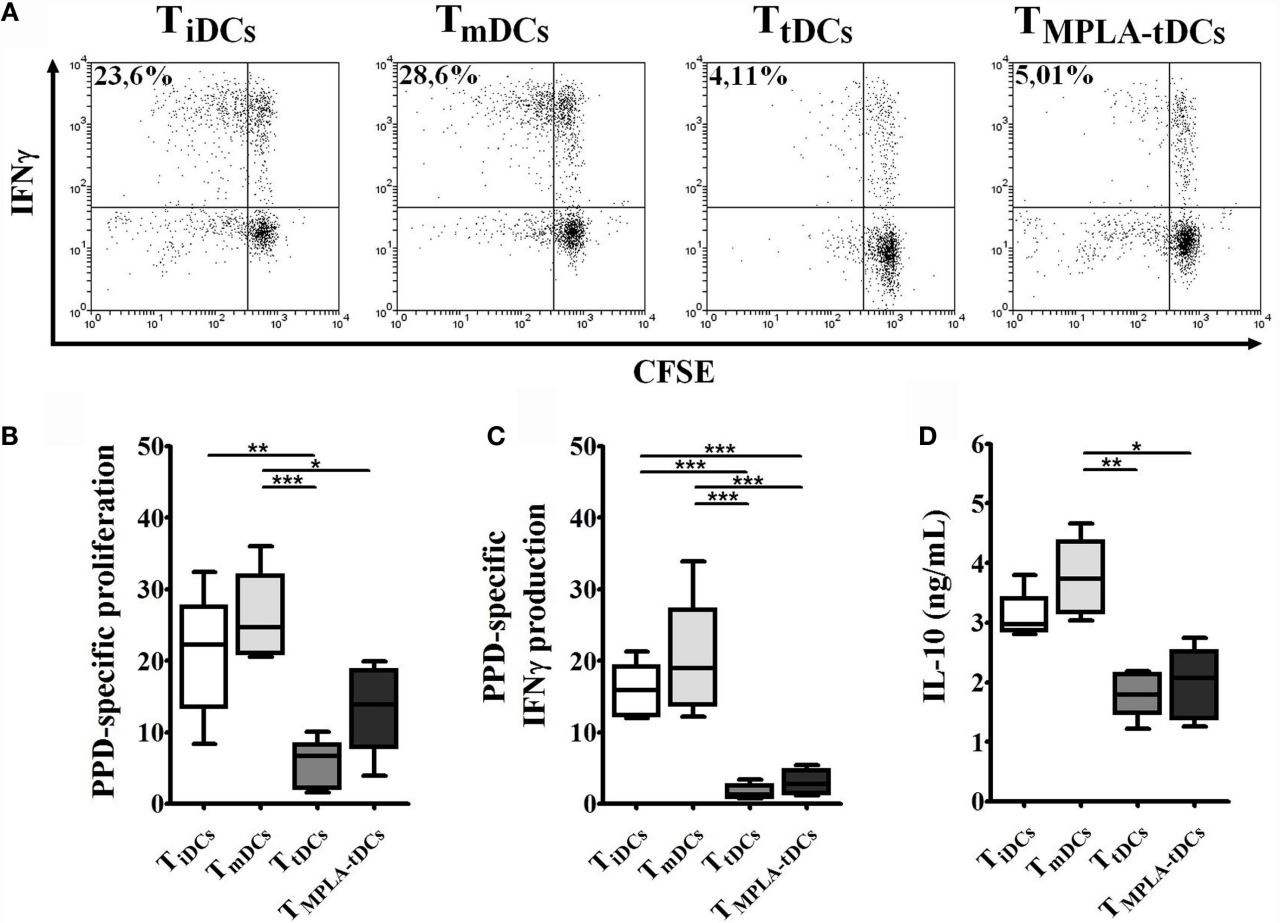
**MPLA-tDCs render memory CD4^+^ T cells to be hyporesponsive to restimulation**. For restimulation assays, memory CD4^+^ T cells were primed with PPD-loaded DC subsets and then were recovered, washed, labeled with CFSE, and restimulated with unpulsed or PPD-pulsed mDCs. Data were normalized by subtracting values of unpulsed conditions to antigen-specific conditions. **(A)** Representative dot plots of IFN-γ expression from six independent experiments are shown. **(B)** Proliferation of restimulated primed-CD4^+^ T cells was determined by CFSE dilution through flow cytometry and graphic representation of normalized data of the percentage of CFSE^low^ in CD4^+^ T cells is shown. **(C)** IFN-γ expression was detected intracellularly and analyzed by flow cytometry. Graphic representation of normalized data of the percentage of IFN-γ producing proliferating CD4^+^ T cells (CFSE^low^) is shown. **(D)** IL-10 secretion levels from mDC/primed-T cell co-culture supernatants were measured using ELISA. For **(B–D)**, statistical differences were calculated using Friedman test (for non-parametric data) or one-way ANOVA (for normally distributed data) followed by Duns or Tukey post-test, respectively (*P < 0.05; **P < 0.01; ***P < 0.001).

To evaluate if the first encounter with MPLA-tDCs affects CD4^+^ T cell function, we recovered CD4^+^ T lymphocytes from co-cultures with different DC subtypes and re-challenged them with autologous unpulsed or PPD-pulsed mDCs. A reduced proliferative response and proinflammatory cytokines production were evidenced when recovered CD4^+^ T cells were co-cultured with unpulsed mDCs in comparison to pulsed conditions (data not shown). Memory CD4^+^ T cells recovered from co-cultures with tDCs and MPLA-tDCs exhibited a reduced proliferation and IFN-γ production in response to restimulation with PPD-pulsed mDCs, compared to memory CD4^+^ T cells derived from co-cultures with iDCs or mDCs (Figures 3A–C). However, previous contact to MPLA-tDCs or tDCs also diminished IL-10 secretion by memory CD4^+^ T cells compared to those previously stimulated by mDCs (Figure 3D). No significant differences were evident in the IL-17 expression by memory CD4^+^ T cells primed by different DC subtypes (data not shown).

**Figure 4:**
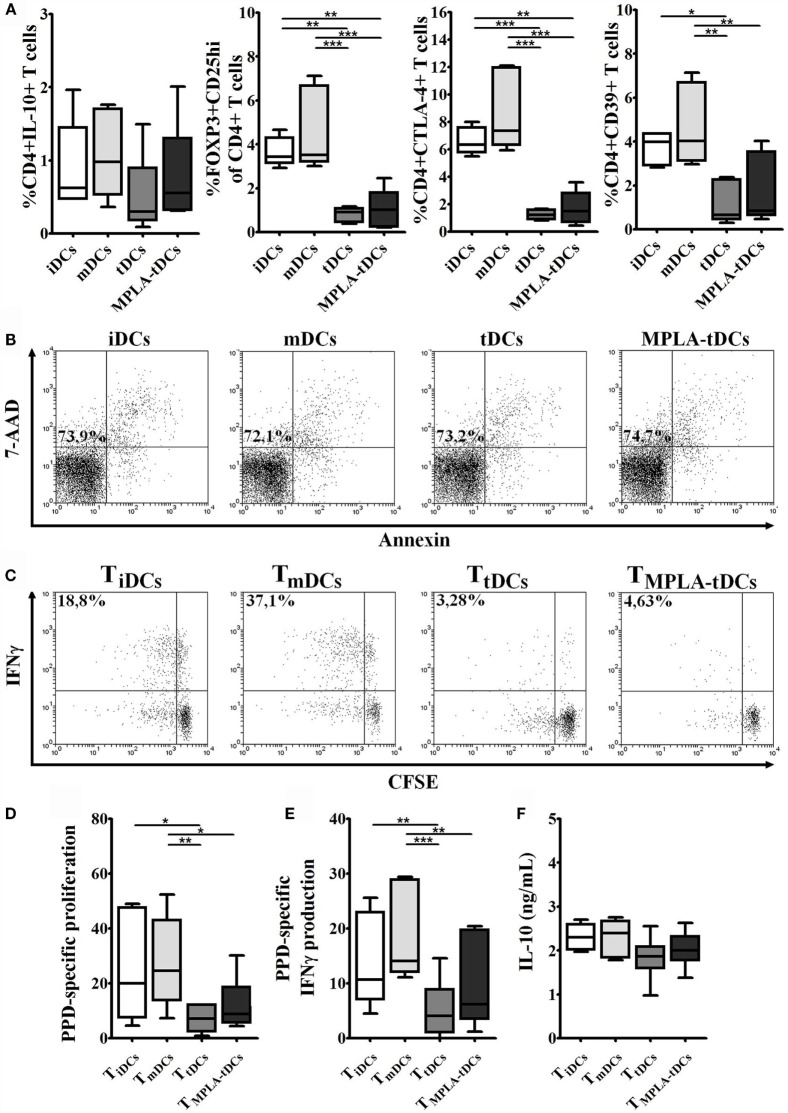
**Naive CD4^+^ T cells primed by MPLA-tDCs do not exhibit a regulatory phenotype and become hyporesponsive to restimulation**. MPLA-tDCs ability to induce phenotypic regulatory properties on naive CD4^+^ T cells after co-culturing was evaluated. **(A)** Expression of IL-10, FOXP3 in combination to CD25, CTLA-4, and CD39 was evaluated by flow cytometry. Graphic analyses of MFI measurements are expressed as box-and-whiskers plot from minimum to maximum values (*n* = 5). Statistical differences were calculated using Friedman test (for non-parametric data) or one-way ANOVA (for normally distributed data) followed by Duns or Tukey post-test, respectively (*P < 0.05; **P < 0.01; ***P < 0.001). **(B)** Representative dot plots of cell viability measured after 6 days of co-culture and expressed as the percentage of annexin-V and 7-AAD-negative cells are shown. For restimulation assays, naive CD4^+^ T cells were primed with PPD-loaded DC subsets and then were recovered, washed, labeled with CFSE, and restimulated with unpulsed or PPD-pulsed mDCs. Data were normalized by subtracting values of unpulsed conditions to antigen-specific conditions. **(C)** Representative dot plots of IFN-γ expression from six independent experiments are shown. **(D)** Proliferation of restimulated primed-CD4^+^ T cells was determined by CFSE dilution through flow cytometry, and graphic representation of normalized data of the percentage of CFSE^low^ in CD4^+^ T cells is shown. **(E)** IFN-γ expression was detected intracellularly and analyzed by flow cytometry. Graphic representation of normalized data of the percentage of IFN-γ producing proliferating CD4^+^ T cells (CFSE^low^) is shown. **(F)** IL-10 secretion levels from mDC/primed-T cell co-culture supernatants were measured using ELISA. For **(D–F)**, statistical differences were calculated using one-way ANOVA followed by Tukey post-test (*P < 0.05; **P < 0.01; ***P < 0.001).

Naive CD4^+^ T cells that were initially co-cultured with tDCs or MPLA-tDCs exhibited lower proliferation and IFN-γ production toward restimulation with PPD-pulsed mDCs than those that were previously primed by PPD-loaded mDCs (Figures 4C–E). The response of tDCs-primed naive CD4^+^ T cells was also significantly lower than that of iDCs-primed naive CD4^+^ T cells (Figures 4D,E). In contrast, IL-10 secretion levels did not differ between CD4^+^ T cells that were previously primed by iDCs, mDCs, tDCs, or MPLA-tDCs (Figure 4F). No significant differences were evident in the IL-17 expression by memory CD4^+^ T cells primed by different DC subtypes (data not shown).

### MPLA-tDCs Endow Naive CD4^+^ T Cells with the Ability to Suppress Th1 and Th17 Responses

**Figure 5:**
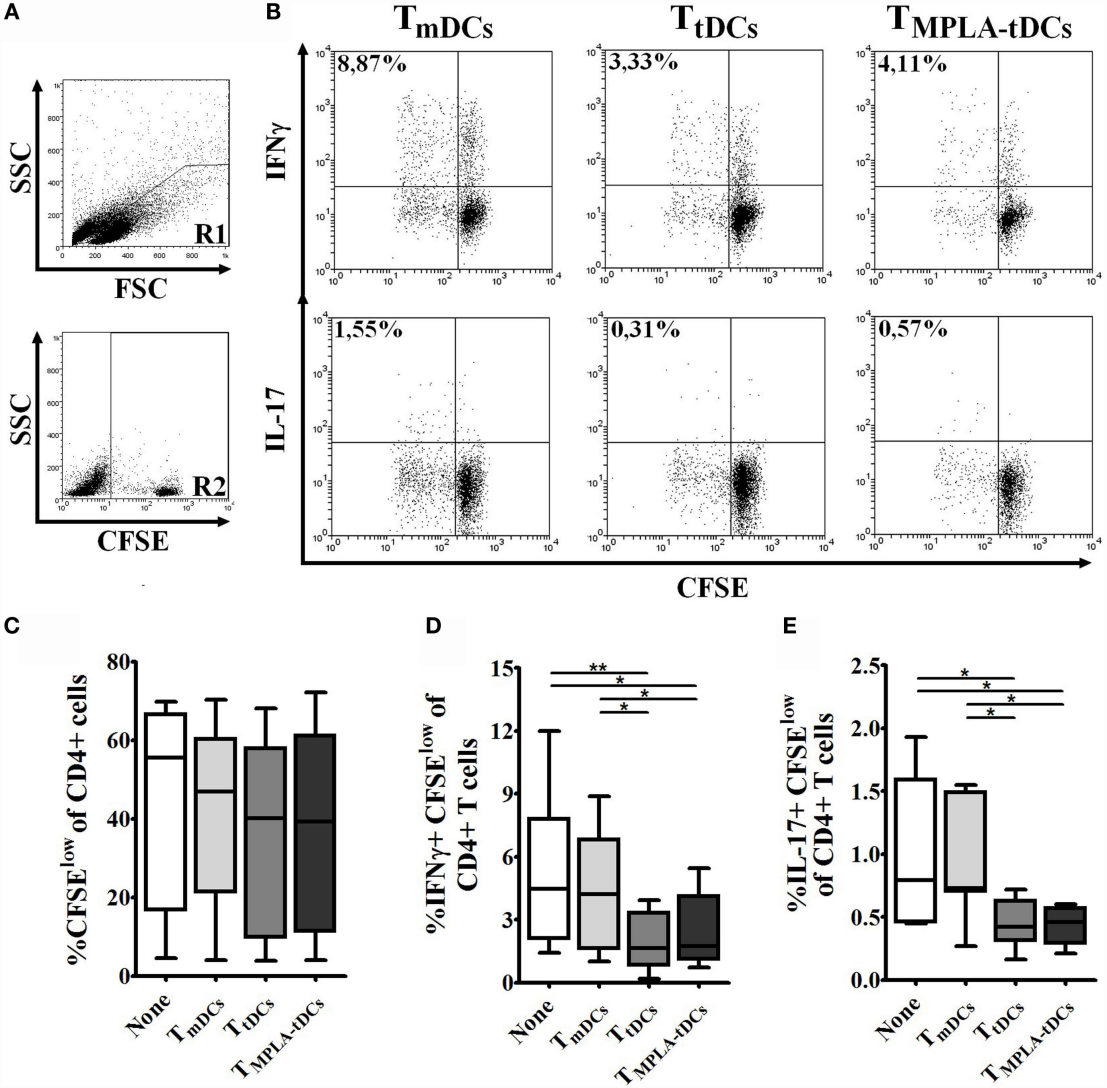
**MPLA-tDCs endow naive CD4^+^ T cells with the ability to suppress Th1 and Th17 responses**. To evaluate the ability of MPLA-tDCs to induce functional regulatory properties on naive CD4^+^ T cells, these cells were primed with PPD-loaded DC subsets and then were recovered, washed, and added to a co-culture of CFSE-labeled responder CD4^+^ T cells and PPD-pulsed mDCs. Responder CD4^+^ T cells co-cultured with mDCs and without the addition of primed-naive CD4^+^ T cells were used as control. **(A)** Gate strategy is shown: R1 = lymphocytes region, R2 = CFSE + population. **(B)** Representative dot plots of IFN-γ (top) and IL-17 (bottom) expression from seven independent experiments are shown. **(C)** Proliferation of responder CD4^+^ T cells in presence of primed-naive CD4^+^ T cells was determined by CFSE dilution through flow cytometry, and graphic representation of the percentage of CFSE^low^ in CD4^+^ T cells is shown. **(D)** IFN-γ and **(E)** IL-17 expression were detected intracellularly and analyzed by flow cytometry. Graphic representation of the percentage of IFN-γ and IL-17 producing proliferating CD4^+^ T cells (CFSE^low^) is shown. For **(C–E)**, statistical differences were calculated using one-way ANOVA followed by Tukey post-test (*P < 0.05; **P < 0.01).

Having shown that MPLA-tDCs render naive CD4^+^ T cells hyporesponsive to second stimulation by mDCs, we wanted to investigate whether MPLA-tDCs also confer the ability to suppress effector T cell responses. Therefore, we co-cultured naive CD4^+^ T cells, previously primed by PPD-pulsed DCs, with autologous CFSE-labeled responder CD4^+^ T cells and PPD-pulsed mDCs (Figure 5A). The addition of previously primed naive CD4^+^ T cells to co-cultures did not affect responder cell proliferation (Figure 5C). However, IFN-γ and IL-17 production by responder CD4^+^ T cells was markedly reduced in the presence of naive CD4^+^ T cells that were previously primed by MPLA-tDCs or tDCs in comparison to co-cultures that received mDC-primed naive CD4^+^ T cells and co-cultures performed in absence of primed-naive CD4^+^ T cells (Figures 5B,D,E), indicating that MPLA-tDCs confer suppressive features to these cells.

## Discussion

Cell therapy based on tolerogenic DCs (tDCs) has become a promising alternative for the treatment of T cell-mediated autoimmune pathologies (31). Our group has recently developed a shortened 5-day protocol for generation of alternatively activated tDCs, using Dex for modulation and MPLA as activating stimulus (26). The present study confirms the ability of these MPLA-tDCs to act at different levels of an immune response, preventing proinflammatory naive and memory CD4^+^ T cell responses. Moreover, MPLA-tDCs do not only render naive and memory T cells hyporesponsive to further stimulation by DCs but also imprint regulatory functions such as the suppression of Th1 and Th17 effector responses.

Consistent with our previous work, we observed low expression levels of costimulatory molecules, such as CD86, CD80, and CD83, in both tDCs and MPLA-tDCs, indicating that MPLA treatment does not affect the induction of a tolerogenic phenotype (26). Costimulatory ligands provide the “second signal” for T cell activation (32, 33), and their reduced expression on DCs is associated with a decreased capacity to initiate T cell responses and the promotion of tolerance through induction of T cell anergy and/or differentiation into Tregs (8).

As antigen capture is crucial to the presentation of exogenous antigen by DCs, we evaluated the effect of Dex and MPLA treatment on the uptake of DX-FITC particles, which are mainly ingested *via* receptor-mediated endocytosis through mannose receptor (34). DCs that did not receive MPLA treatment (iDCs and tDCs) presented higher uptake of DX-FITC than MPLA-treated DCs (mDCs and MPLA-tDCs). In accordance with the previous reports, Dex treatment of DCs even enhanced the rate of mannose receptor-mediated endocytosis as compared to iDCs (35). As antigen uptake is a hallmark of immature DCs and decreases upon maturation (34), antigen loading has to be accomplished prior to application of maturation stimuli such as MPLA. Therefore, in our experimental model, mDCs and MPLA-tDCs were exposed to PPD antigen 4 h before addition of MPLA, enabling sufficient antigen uptake.

It has been reported that activation of tDCs by adding TLR ligands or proinflammatory cytokines increases the antigen-presenting capacity, which is indispensable for antigen-specific therapeutic approaches (28). Applying MPLA as activation stimulus, we achieved HLA-DR levels on MPLA-tDCs similar to their mature counterparts, suggesting that MPLA-tDCs are able to present antigen. Using an hCII-specific murine T cell hybridoma derived from HLA-DR1 transgenic mice (36), we confirmed that MPLA-tDCs are able to present antigens efficiently, similar to mDCs and more efficient than tDCs.

The hitherto described tolerogenic features of MPLA-tDCs, in addition to the previously reported (26), make them a promising candidate for immunotherapy of autoimmune diseases. However, more sophisticated *in vitro* and *in vivo* studies are required to validate their suitability for the transfer to clinical application. To attend this requirement, we evaluated the ability of MPLA-tDCs to modulate antigen-specific naive and memory CD4^+^ T cell responses using PPD as model antigen. MPLA-tDCs were poor inducers of proliferation and IFN-γ and IL-17 production in PPD-specific memory CD4^+^ T cells. This effect was not associated with an induction of apoptosis, suggesting rather reprograming than deletion of antigen-specific memory CD4^+^ T cells (27). We confirmed this hypothesis of “reprograming” by a restimulation assay, subjecting memory CD4^+^ T cells, recovered from co-cultures with different DC subtypes, to a second stimulation with PPD-pulsed mDCs. Only memory CD4^+^ T cells that had previously contact to mDCs or iDCs proliferated and produced IFN-γ in response to the second stimulus, while memory CD4^+^ T cells derived from co-cultures with MPLA-tDCs or tDCs remained completely unresponsive to antigen-specific stimulation.

Interestingly, naive CD4^+^ T cells did not proliferate after first encounter with different DC subsets. It is conceivable that naive CD4^+^ T cells received an initial “imprinting” by the respective DC subset but need a prolonged time of stimulation or a second encounter with DCs to develop a proliferative response. However, an “imprinting” of naive CD4^+^ T cells by tDCs and MPLA-tDCs confers hyporesponsiveness in terms of proliferation and IFN-γ production toward second stimulation with PPD-pulsed mDCs.

Unlike other studies, in which priming with Dex-treated DCs increases the number of IL-10-producing cells both *in vivo* and *in vitro* (37, 38), in our model, there were no significant differences in IL-10 expression between naive CD4^+^ T cells primed with different DC subsets. This could be due to the fact that repetitive rounds of priming with tDCs are necessary to modulate T cells properties, as reported by Unger and colleagues (39). This would explain why we were not able to observe a typical Treg phenotype on naive CD4^+^ T cells primed by MPLA-tDCs or tDCs. It has to be considered that the analyzed markers are not exclusively expressed by Tregs but also by activated T cells (31). Thus, it is essential to unravel markers that specifically define and distinguish distinct T cell populations with regulatory properties.

The observed “reprograming” of antigen-specific naive and memory CD4^+^ T cells by MPLA-tDCs to a stable hyproresponsive state is in accordance with a study of Woltman and colleagues (29) and suggests that in the context of autoimmunity, transferred autologous MPLA-tDCs would be able to silence autoreactive T cell responses, either at their initiation, preventing the differentiation of autoantigen-specific naive CD4^+^ T cells toward proinflammatory Th1 or Th17 cells, or by turning off already developed effector T cell responses.

Anergy induction appears to be a promising strategy to specifically target pathologic T cell responses without generating undesired side effects. Several studies have demonstrated that the induction of CD4^+^ T cell anergy efficiently controls responses against disease-associated autoantigens in patients with autoimmune pathologies *in vitro* (24, 40, 41), preventing disease progression *in vivo* (14, 42). Furthermore, it has been reported that hyporesponsive CD4^+^ T cells can also acquire suppressive capacities, supporting their role in the restoration of tolerance (43, 44).

Likewise, an important finding of this work is that despite the lack of characteristic Treg markers, naive CD4^+^ T cells primed in tolerogenic conditions, i.e., the presence of MPLA-tDCs or tDCs, acquired immunomodulatory functions, inhibiting the polarization of responder CD4^+^ T cells to Th1 and Th17 profiles. There are three possible mechanisms: first, we are generating hyporesponsive CD4^+^ T cells with suppressive capacities, as described previously (43, 44). Second, naive CD4^+^ T cells primed by MPLA-tDCs or tDCs might compete with effector CD4^+^ T cells for the contact to mDCs, thereby preventing the development of an immunological synapse and an efficient T cell activation. Third, MPLA-tDC- or tDC-primed naive CD4^+^ T cells might express yet unknown mediators that either inhibit DC function (45) or render effector T cells anergic (46).

In this work, we investigated different scenarios that antigen-loaded MPLA-tDCs might encounter *in vivo*, e.g., when transferred into a patient with autoimmune disease, including naive autoantigen-specific CD4^+^ T cells and mDCs in the lymph node, or autoreactive memory CD4^+^ T cells and inflammatory DCs in inflamed tissues. We showed that MPLA-tDCs were able to reprogram memory CD4^+^ T cells to become anergic toward second stimulation and to imprint regulatory functions on naive CD4^+^ T cells. Although further *in vivo* studies are required, our results provide important evidence that MPLA-tDCs are suitable immunotherapeutics for the establishment of antigen-specific tolerance in autoimmunity and transplantation.

## Author Contributions

JA and DC participated in the design of the study. JM, KS, and BP participated in data acquisition and analysis. JM prepared the manuscript. JA, DC, KS, BP, and CH participated in data interpretation and manuscript revision.

## Conflict of Interest Statement

The authors declare that the research was conducted in the absence of any commercial or financial relationships that could be construed as a potential conflict of interest.

## References

[1] Glück T Kiefmann B Grohmann M Falk W Straub RH Schölmerich J. Immune status and risk for infection in patients receiving chronic immunosuppressive therapy. J Rheumatol (2005) 32(8):1473–80. 16078322

[2] Gutierrez-Dalmau A Campistol JM. Immunosuppressive therapy and malignancy in organ transplant recipients: a systematic review. Drugs (2007) 67(8):1167–98. 10.2165/00003495-200767080-00006 17521218

[3] van Duivenvoorde L van Mierlo G Boonman Z Toes R. Dendritic cells: vehicles for tolerance induction and prevention of autoimmune diseases. Immunobiology (2006) 211(6–8):627–32. 10.1016/j.imbio.2006.05.014 16920501

[4] Banchereau J Steinman R Dendritic cells and the control of immunity. Nature (1998) 392(6673):245–52. 10.1038/32588 9521319

[5] Moser M Dendritic cells in immunity and tolerance – do they display opposite functions? Immunity (2003) 19(1):5–8. 10.1016/S1074-7613(03)00182-1 12871634

[6] Naranjo-Gomez M Raich-Regue D Onate C Grau-Lopez L Ramo-Tello C Pujol-Borrell R Comparative study of clinical grade human tolerogenic dendritic cells. J Transl Med (2011) 9(1):89. 10.1186/1479-5876-9-89 21658226 PMC3141500

[7] Torres-Aguilar H Aguilar-Ruiz SR González-Pérez G Munguía R Bajaña S Meraz-Ríos MA Tolerogenic dendritic cells generated with different immunosuppressive cytokines induce antigen-specific anergy and regulatory properties in memory CD4+ T cells. J Immunol (2010) 184(4):1765–75. 10.4049/jimmunol.0902133 20083662

[8] Steinman R Hawiger D Nussenzweig M. Tolerogenic dendritic cells. Annu Rev Immunol (2003) 21:685–711. 10.1146/annurev.immunol.21.120601.141040 12615891

[9] Salazar L Aravena O Abello P Escobar A Contreras-Levicoy J Rojas-Colonelli N Modulation of established murine collagen-induced arthritis by a single inoculation of short-term lipopolysaccharide-stimulated dendritic cells. Ann Rheum Dis (2008) 67(9):1235–41. 10.1136/ard.2007.072199 18056756

[10] Popov I Li M Zheng X San H Zhang X Ichim T Preventing autoimmune arthritis using antigen-specific immature dendritic cells: a novel tolerogenic vaccine. Arthritis Res Ther (2006) 8(5):R141. 10.1186/ar2031 16911769 PMC1779442

[11] van Duivenvoorde L Han W Bakker A Louis-Plence P Charbonnier L-M Apparailly F Immunomodulatory dendritic cells inhibit Th1 responses and arthritis via different mechanisms. J Immunol (2007) 179(3):1506–15. 10.4049/jimmunol.179.3.1506 17641016

[12] Stoop J Harry R von Delwig A Isaacs J Robinson J Hilkens C. Therapeutic effect of tolerogenic dendritic cells in established collagen-induced arthritis is associated with a reduction in Th17 responses. Arthritis Rheum (2010) 62(12):3656–65. 10.1002/art.27756 20862679

[13] Adorini L Penna G Giarratana N Uskokovic M Tolerogenic dendritic cells induced by vitamin D receptor ligands enhance regulatory T cells inhibiting allograft rejection and autoimmune diseases. J Cell Biochem (2003) 88(2):227–33. 10.1002/jcb.10340 12520519

[14] Ferreira G Gysemans C Demengeot J da Cunha J Vanherwegen A Overbergh L 1,25-dihydroxyvitamin D3 promotes tolerogenic dendritic cells with functional migratory properties in NOD mice. J Immunol (2014) 192(9):4210–20. 10.4049/jimmunol.1302350 24663679

[15] Chorny A Gonzalez-Rey E Fernandez-Martin A Pozo D Ganea D Delgado M. Vasoactive intestinal peptide induces regulatory dendritic cells with therapeutic effects on autoimmune disorders. Proc Natl Acad Sci U S A (2005) 102(38):13562–7. 10.1073/pnas.0504484102 16150720 PMC1224633

[16] Giannoukakis N Phillips B Finegold D Harnaha J Trucco M. Phase I (safety) study of autologous tolerogenic dendritic cells in type 1 diabetic patients. Diabetes Care (2011) 34(9):2026–32. 10.2337/dc11-0472 21680720 PMC3161299

[17] Benham H Nel H Law S Mehdi A Street S Ramnoruth N Citrullinated peptide dendritic cell immunotherapy in HLA risk genotype-positive rheumatoid arthritis patients. Sci Transl Med (2015) 7(290):290ra87. 10.1126/scitranslmed.aaa9301 26041704

[18] Bell GM Anderson AE Diboll J Reece R Eltherington O Harry RA Autologous tolerogenic dendritic cells for rheumatoid and inflammatory arthritis. Ann Rheum Dis (2016) 1–8. 10.1136/annrheumdis-2015-208456 27117700 PMC5264217

[19] Sallusto F Lanzavecchia A. Efficient presentation of soluble antigen by cultured human dendritic cells is maintained by granulocyte/macrophage colony-stimulating factor plus interleukin 4 and downregulated by tumor necrosis factor alpha. J Exp Med (1994) 179(4):1109–18. 10.1084/jem.179.4.1109 8145033 PMC2191432

[20] Zheng X Suzuki M Ichim T Zhang X Sun H Zhu F Treatment of autoimmune arthritis using RNA interference-modulated dendritic cells. J Immunol (2010) 184(11):6457–64. 10.4049/jimmunol.0901717 20435931

[21] Henry E Desmet C Garzé V Fiévez L Bedoret D Heirman C Dendritic cells genetically engineered to express IL-10 induce long-lasting antigen-specific tolerance in experimental asthma. J Immunol (2008) 181(10):7230–42. 10.4049/jimmunol.181.10.7230 18981145

[22] Morita Y Yang J Gupta R Shimizu K Shelden E Endres J Dendritic cells genetically engineered to express IL-4 inhibit murine collagen-induced arthritis. J Clin Invest (2001) 107(10):1275–84. 10.1172/jci11490 11375417 PMC209294

[23] Sochorová K Budinský V Rozková D Tobiasová Z Dusilová-Sulková S Spísek R Paricalcitol (19-nor-1,25-dihydroxyvitamin D2) and calcitriol (1,25-dihydroxyvitamin D3) exert potent immunomodulatory effects on dendritic cells and inhibit induction of antigen-specific T cells. Clin Immunol (2009) 133(1):69–77. 10.1016/j.clim.2009.06.011 19660988

[24] Harry R Anderson A Isaacs J Hilkens C. Generation and characterisation of therapeutic tolerogenic dendritic cells for rheumatoid arthritis. Ann Rheum Dis (2010) 69(11):2042–50. 10.1136/ard.2009.126383 20551157 PMC3002758

[25] Adikari S Pettersson A Soderstrom M Huang YM Link H. Interleukin-10-modulated immature dendritic cells control the proinflammatory environment in multiple sclerosis. Scand J Immunol (2004) 59(6):600–6. 10.1111/j.1365-3083.2004.01453.x 15182256

[26] García-González P Morales R Hoyos L Maggi J Campos J Pesce B A short protocol using dexamethasone and monophosphoryl lipid A generates tolerogenic dendritic cells that display a potent migratory capacity to lymphoid chemokines. J Transl Med (2013) 11:128. 10.1186/1479-5876-11-128 23706017 PMC3674980

[27] Anderson AE Sayers BL Haniffa MA Swan DJ Diboll J Wang X-N Differential regulation of naïve and memory CD4+ T cells by alternatively activated dendritic cells. J Leukoc Biol (2008) 84(1):124–33. 10.1189/jlb.1107744 18430785 PMC2504714

[28] Anderson AE Swan DJ Sayers BL Harry RA Patterson AM von Delwig A LPS activation is required for migratory activity and antigen presentation by tolerogenic dendritic cells. J Leukoc Biol (2009) 85(2):243–50. 10.1189/jlb.0608374 18971286 PMC2700018

[29] Woltman A van der Kooij S de Fijter J van Kooten C. Maturation-resistant dendritic cells induce hyporesponsiveness in alloreactive CD45RA+ and CD45RO+ T-cell populations. Am J Transplant (2006) 6(11):2580–91. 10.1111/j.1600-6143.2006.01520.x 16952295

[30] von Delwig A Altmann D Isaacs J Harding C Holmdahl R McKie N The impact of glycosylation on HLA-DR1-restricted T cell recognition of type II collagen in a mouse model. Arthritis Rheum (2006) 54(2):482–91. 10.1002/art.21565 16447222

[31] Maggi J Schafer C Ubilla-Olguín G Catalán D Schinnerling K Aguillón JC. Therapeutic potential of hyporesponsive CD4(+) T cells in autoimmunity. Front Immunol (2015) 6:488. 10.3389/fimmu.2015.00488 26441992 PMC4585084

[32] Linsley PS Ledbetter JA. The role of the CD28 receptor during T cell responses to antigen. Annu Rev Immunol (1993) 11(1):191–212. 10.1146/annurev.iy.11.040193.001203 8386518

[33] Schwartz RH Costimulation of T lymphocytes: the role of CD28, CTLA-4, and B7/BB1 in interleukin-2 production and immunotherapy. Cell (1992) 71(7):1065–8. 10.1016/s0092-8674(05)80055-8 1335362

[34] Sallusto F Cella M Danieli C Lanzavecchia A. Dendritic cells use macropinocytosis and the mannose receptor to concentrate macromolecules in the major histocompatibility complex class II compartment: downregulation by cytokines and bacterial products. J Exp Med (1995) 182(2):389–400. 10.1084/jem.182.2.389 7629501 PMC2192110

[35] Piemonti L Monti P Allavena P Leone B Caputo A Carlo V. Glucocorticoids increase the endocytic activity of human dendritic cells. Int Immunol (1999) 11(9):1519–26. 10.1093/intimm/11.9.1519 10464173

[36] Canaday D Gehring A Leonard E Eilertson B Schreiber J Harding C T-cell hybridomas from HLA-transgenic mice as tools for analysis of human antigen processing. J Immunol Methods (2003) 281(1–2):129–42. 10.1016/j.jim.2003.07.004 14580887

[37] Roelen DL Schuurhuis DH van den Boogaardt DEE Koekkoek K van Miert PP van Schip JJ Prolongation of skin graft survival by modulation of the alloimmune response with alternatively activated dendritic cells. Transplantation (2003) 76(11):1608–15. 10.1097/01.tp.0000086340.30817.ba 14702533

[38] Vizzardelli C Pavelka N Luchini A Zanoni I Bendickson L Pelizzola M Effects of dexamethasone on LPS-induced activation and migration of mouse dendritic cells revealed by a genome-wide transcriptional analysis. Eur J Immunol (2006) 36(6):1504–15. 10.1002/eji.200535488 16708398

[39] Unger W Laban S Kleijwegt F van der Slik A Roep B. Induction of Treg by monocyte-derived DC modulated by vitamin D3 or dexamethasone: differential role for PD-L1. Eur J Immunol (2009) 39(11):3147–59. 10.1002/eji.200839103 19688742

[40] Raïch-Regué D Grau-López L Naranjo-Gómez M Ramo-Tello C Pujol-Borrell R Martínez-Cáceres E Stable antigen-specific T-cell hyporesponsiveness induced by tolerogenic dendritic cells from multiple sclerosis patients. Eur J Immunol (2012) 42(3):771–82. 10.1002/eji.201141835 22488365

[41] Segovia-Gamboa N Rodríguez-Arellano M Rangel-Cruz R Sánchez-Díaz M Ramírez-Reyes J Faradji R Tolerogenic dendritic cells induce antigen-specific hyporesponsiveness in insulin- and glutamic acid decarboxylase 65-autoreactive T lymphocytes from type 1 diabetic patients. Clin Immunol (2014) 154(1):72–83. 10.1016/j.clim.2014.06.009 24993292

[42] Mansilla M Sellès-Moreno C Fàbregas-Puig S Amoedo J Navarro-Barriuso J Teniente-Serra A Beneficial effect of tolerogenic dendritic cells pulsed with MOG autoantigen in experimental autoimmune encephalomyelitis. CNS Neurosci Ther (2015) 21(3):222–30. 10.1111/cns.12342 25403984 PMC6495243

[43] Steinbrink K CD4+ and CD8+ anergic T cells induced by interleukin-10-treated human dendritic cells display antigen-specific suppressor activity. Blood (2002) 99(7):2468–76. 10.1182/blood.V99.7.2468 11895781

[44] Pletinckx K Vaeth M Schneider T Beyersdorf N Hünig T Berberich-Siebelt F Immature dendritic cells convert anergic nonregulatory T cells into Foxp3(-) IL-10(+) regulatory T cells by engaging CD28 and CTLA-4. Eur J Immunol (2015) 45(2):480–91. 10.1002/eji.201444991 25382658

[45] Graham DB Akilesh HM Gmyrek GB Piccio L Gilfillan S Sim J ITAM signaling in dendritic cells controls T helper cell priming by regulating MHC class II recycling. Blood (2010) 116(17):3208–18. 10.1182/blood-2009-10-250415 20634378 PMC2995352

[46] Ermann J Szanya V Ford GS Paragas V Fathman CG Lejon K. CD4(+)CD25(+) T cells facilitate the induction of T cell anergy. J Immunol (2001) 167(8):4271–5. 10.4049/jimmunol.167.8.4271 11591749

